# Religion and Spirituality in Oncology: An Exploratory Study of the Communication Experiences of Clinicians in Poland

**DOI:** 10.1007/s10943-021-01343-1

**Published:** 2021-07-31

**Authors:** Oliwia Kowalczyk, Krzysztof Roszkowski, Wojciech Pawliszak, Agnieszka Rypel, Szymon Roszkowski, Jolanta Kowalska, Bartosz Tylkowski, Anna Bajek

**Affiliations:** 1 Research and Education Unit for Communication in Healthcare, Department of Cardiac Surgery, Ludwik Rydygier Collegium Medicum in Bydgoszcz Nicolaus Copernicus University in Torun, M. Sklodowskiej Curie St. 9, 85-094 Bydgoszcz, Poland; 2 Department of Oncology, Ludwik Rydygier Collegium Medicum in Bydgoszcz Nicolaus Copernicus University in Torun, Romanowskiej St. 2, 85-796 Bydgoszcz, Poland; 3 Department of Cardiac Surgery, Ludwik Rydygier Collegium Medicum in Bydgoszcz Nicolaus Copernicus University in Torun, M. Sklodowskiej Curie St. 9, 85-094 Bydgoszcz, Poland; 4 Faculty of Linguistics, Kazimierz Wielki Univeristy in Bydgoszcz, Jagiellonska St. 11, 85-067 Bydgoszcz, Poland; 5 Department of Biochemistry and Biogerontology, Ludwik Rydygier Collegium Medicum in Bydgoszcz Nicolaus Copernicus University in Torun, Debowa St. 3, 85-626 Bydgoszcz, Poland; 6 Institute of Plant Protection, National Research Institute, Wegorka St. 20, 60-318 Poznan, Poland; 7 Chemical Unit, Eurecat, Center Tecnològic de Catalunya, Marcellí Domingo s/n, 43007 Tarragona, Spain; 8 Department of Tissue Engineering Chair of Urology and Andrology, Ludwik Rydygier Collegium Medicum in Bydgoszcz Nicolaus Copernicus University in Torun, Karlowicza St. 24, 85-092 Bydgoszcz, Poland

**Keywords:** Communication in healthcare, Cancer care, Religion, Spirituality, Empathy, Oncologists, Radiotherapists

## Abstract

Communication with patients regarding oncology-related aspects is a challenging experience and requires a high level of skill from the interlocutors. The aim of this study was to verify the influence of religion/spirituality in oncological settings from the health professionals’ perspectives in Poland. It assessed the role of religion/spirituality in patient-clinician communication, death or stress self-management, empathy, and breaking bad news skills. Data collection was carried out through a standardized self-administered questionnaire with varying scales. The study cohort consisted of 60 medical practitioners specializing in oncological radiotherapy treatments. It was observed that strategies used for coping with patients’ death, stress reduction, empathy, communication with patients and/or their relatives, or breaking bad news skills, may be gender-specific or may depend on the length of time employed, as well as experience in a cancer-related work environment. This study shows that spirituality and religiousness can support clinicians in managing challenging or negative emotions related to their work in cancer settings. Religiousness and spirituality can also serve as a potential therapeutic strategies for those exposed to patient suffering and death.

## Introduction

Religious/spiritual (R/S) beliefs influence patients’ decisions and are correlated with better quality of life and affect psychological adjustment to challenging daily experiences among clinicians in oncology-related environments (Mishra et al., 2017; Puchalski et al., 2006, 2009). In some studies, R/S are defined as separate measures (Peteet et al., 2019) in some they overlap (Phelps et al., 2012), while others still emphasize religion over spirituality due to reliable measurement metrics (Koenig, 2008; Pargament, 2013). In our research approach, we define both as phenomena supporting patients’ oncological experiences, as well as resources providing equanimity and self-care for clinicians experiencing exposure to dying, grief, and suffering. Thus, we relinquished further differentiation, and in the further descriptions, we use the general term faith.

As cancer is the leading mortality cause worldwide (Siegel et al., 2019), it follows that oncologists are much more exposed to death and suffering compared to clinicians in other fields. Due to the technological advancements, personalized therapies, and a resulting increase in survival rates (Li et al., 2016), the relationships between oncologists and their patients remain more prolonged, and therefore more demanding and intense from a psychological point of view. R/S are therefore essential elements and play a significant support role in the care of oncological patients. A study conducted in 13 U.S. hospitals among all pediatric oncology faculty by Ecklund, et al. shows that 85% of the survey participants depicted themselves as spiritual. Of those, 52.7% consider their spiritual or religious beliefs to determine their interactions with patients (Ecklund et al., 2007). Another U.S. study performed among academic faculty by the same team similarly reported that 58.6% of the physicians described their spiritual or religious beliefs as influential regarding interactions with their patients (Catlin et al., 2008). Aspect of coping with patients’ death was represented in yet another study performed in three adult oncological centers by Granek, et al. The results depicted various ways in which oncologists developed spiritual and religious interventions for managing emotions associated with their patients’ deaths (Granek et al., 2016). Even though, as described above, S/R are perceived as positive factors influencing clinicians’ communication skills in oncology context (Phelps et al., 2012), such conversations are not common among professionals. This may result from the fact that spiritual or religious issues conversations are considered advanced communication skills, and such are rarely integrated into patient communication curricula (Balboni et al., 2013; Ford et al., 2012; Todres et al., 2005).

Considering the above, our study aimed to verify the influence of faith in oncological settings from the health professionals’ perspectives.

## Material and Methods

### Study Design and Population

The population surveyed consisted of Catholic respondents clinical oncologists specializing in radiotherapy (*N* = 60). In the form of an anonymous questionnaire, the survey was conducted over a three month period between November 2020 and February 2021.

The survey assessed demographic data, including sex, age, place of residence, length of employment, and aspects of faith regarding challenging life and death concerns related to oncological context. It also questioned its aspects of communication with patients and their relatives, stress and death self-management, empathy enhancement, and breaking bad news experiences.

### Data Collection Procedures and Analysis

Data collection was carried out through a standardized self-administered questionnaire with varying scales, depending on questions. To gather socio-demographic data, we created our own part of the survey. For assessing the essence and the practice of prayer, we used 5-point Likert scale. For R/S investigation, we used a yes/no questionnaire. Evaluation of benefits of faith and its impact on communication with patients and their families was developed by a research team that also included an oncologist and psycho oncologist, theologian, linguist, and social sciences researcher. Data were analyzed using the software IBM SPSS. Both frequency statistics and non-parametric Pearson's Chi-square test for independent groups (for non-measurable characteristics) were used to determine the correlation between variables.

### Ethical Considerations

All procedures performed in the study involving human participants were under the ethical standards of the Institutional Research Committee (*Bioethical Committee of Ludwik Rydygier Collegium Medicum in Bydgoszcz, Nicolaus Copernicus University in Torun, Poland*)*.* The survey was anonymous and did not require providing any personal data.

## Results

The study group (Table 1) consisted of clinical oncologists specializing in radiotherapy treatments, 73.3% of whom were women, and 26.7% of whom were men, and a majority of the study population declared living in cities over 100 000 inhabitants (86.7%). The largest group of respondents (53.3%) had been working as oncologists between 11 and 20 years (33.3% between 16–20 years and 20% between 11–15 years of hospital employment), and the majority (53.3%) were in the middle-aged cohort (41–50).

**Table 1:** Participant demographic characteristics

Characteristics	Specification	%	*n* = 60
Sex	female	73.3%	44
	male	26.7%	16
Age	30–40	20%	12
	41–50	53.3%	32
	51–60	20%	12
	> 60	6.7%	4
Years of hospital employment	1–5 years	10%	6
	6–10 years	6.7%	4
	11–15 years	20%	12
	16–20 years	33.3%	20
	21–25 years	16.7%	10
	26–30 years	10%	6
	31–35 years	3.3%	2
Place of residence	Village	0%	0
	City < 50 thousand inhabitants	0%	0
	City < 100 thousand inhabitants	0%	0
	City < 250 thousand inhabitants	13.3%	8
	City > 250 thousand inhabitants	86.7%	52
Religious affiliation	Roman Catholic	100%	60

**Fig. 1:**
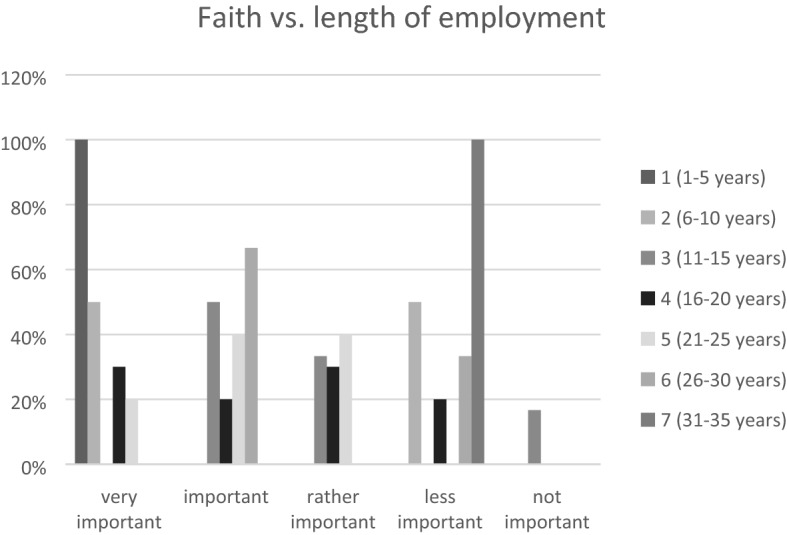
Faith vs. length of employment

The essence of faith concerning the length of employment at their institution (Fig. 1) was declared by 57% of respondents as very important or important. The studied intervals were five years. For those with the shortest employment length (1–5 years) faith was very important (100%), whereas for those with the most extended employment (31–35 years) it was less important (100% in the group between 31 and 35 years of employment).

In the area of changes resulting from working in oncology settings, the obtained results differed significantly between the employment in oncology settings and prior to it. The strengthening importance of faith after employment in oncology settings was declared by 100% of respondents classified by length of employment in groups 2, 4, 6, and 7, as shown in Fig. 1. Regarding the distribution by gender, a similar percentage reported that faith was very important (27% female and 25% male respondents). Considering the overall distribution of the results obtained, faith was more important for women (59% women and 50% men in level 1 and 2 Likert scale).

**Fig. 2:**
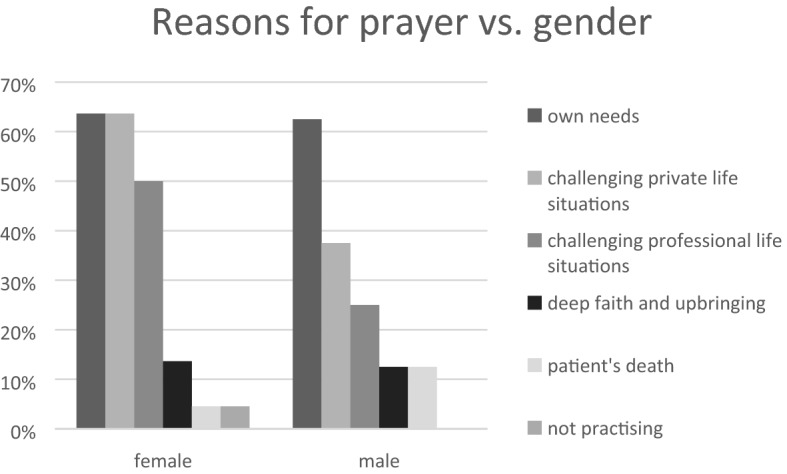
Reasons for prayer vs. gender

As shown in Fig. 2, the study population revealed three main reasons for praying: own needs, challenging encounters in private life, and challenging encounters in professional situations. A similar percentage of women and men pray out of their own needs (64% vs. 63%, respectively). Half of the responding women pray when managing difficult encounters in professional life (50%), while only 25% of men feel such a need. As far as difficulties in private life, most women seek support in prayer (64%), while only 38% of men find solace in such practices.

Faith is also a factor that reduces work-related stress (declared by 77% men, 88% women, respectively) and influences empathy. Within the length of employment categories, faith was important and helped in stress self-management, especially in the groups with the shortest and longest employment periods, as well as in the fifth group according to the set intervals shown in Fig. 1 (100% in the groups with the employment years of 1–5, 21–25, and 31–35, respectively). As far as empathy is concerned, 59% of women and 63% of men declared its impact on their empathy level and skills (100% in the group whose employment length ranges between 21 and 25 years).

In the patient-oncologist communication part of the questionnaire, we investigated how faith impacts challenging conversations with patients and/or their relatives, especially with respect to death or when breaking bad news. Regarding everyday communication experiences with patients, half of the respondents considered faith an influential factor (55% female and 50% male respondents, respectively). Also, half of the responding women declared it helpful in communicating with their patients’ relatives, but only 38% of the responding men considered it as an influential agent. Similar results were obtained as far as breaking bad news is concerned, faith here was seen as a helpful factor by 38% of men and 55% of women. A religion-centered approach serves as a more valuable communication component for women professionals. Its impact, however, increases in managing experiences related to patients’ death (64% of women declared its effect as influential, and so did 50% of the surveyed men). The questionnaire results presented above show an interesting coincidence in relation to the employment time. According to those participants of our research who declared over 25 years of working in oncological settings, faith was not the constituent of communicating unfavorable information, coping with patients’ death, nor facilitating conversations with patients and their relatives. Perhaps according to these individuals, the component playing the most significant influence on the aforementioned elements of communication with patients is their work experience.

## Discussion

R/S beliefs remain increasingly significant in oncology context. The recent years research data have revealed that religious beliefs and spiritual practices are strongly associated with such aspects as: ability to cope with patient’s death, work-related stress reduction, empathy, communication with patients, or breaking bad news skills. Hence, the significance of spirituality or religiousness in clinical practice in oncology settings has been highlighted. Spirituality, in general terms, is often addressed as the quest for a “higher sense” referring to religion or belief in God (Kowalczyk et al., 2020; Mishra et al., 2017). It may be conducted or performed through art, meditation practices, or communing with nature; however, regardless of the means of implementation, it provides a strategy for adjustment in cancer-related work environment.

In our research, we studied the role of spirituality or religious beliefs, which were referred to collectively as faith in the results and analysis section, among a population of clinicians specializing in oncological radiotherapy treatments. The analysis of the survey conducted in one of the biggest oncology centers in Poland, employing 222 doctors specializing in oncology, showed that strategies used for coping with patient’s death, stress reduction, empathy, communication with patients and/or their relatives, or breaking bad news skills, may be gender-specific or may depend on the length of time employed, as well as experience in cancer-related work environment. This is consistent with worldwide research, such as one analysis conducted in 192 countries by Pew Research Center, which proved that women tend to declare faith or spirituality importance and guidance as supportive in the face of challenging situations (Forlenza & Vallada, 2018; Kowalczyk et al., 2020; Li et al., 2016; Peteet et al., 2019). Such attitude is presumably due to psychological disparities between women and men, which may also result in contrasting perceptions within the metaphysical sphere. As our research results show, women tend to be more religious, and it is not necessarily work-related dependency. A majority of the surveyed women declared that their faith did not strengthen due to their working in oncological settings. 50% of female participants indicated faith as an important element of coping with difficult situations in their private lives, 64% in work-related challenges. As far as male participants are concerned, the results were 25% and 38%, respectively.

Oncologists’ job inevitably involves stress due to the emotional impact of unfavorable work circumstances, including difficult ethical decisions or patients’ high mortality. Our study also analyzed the aspect of work-related stress and faith as a contributing factor to its reduction. Faith was reported as an essential self-management strategy factor by 88% of female and 77% of male respondents and was exceptionally high in the shortest and the longest employment time groups.

Working in such a complex field as oncology is fraught with many challenges but requires clinicians to have an appropriate approach in dealing with patients to ensure holistic care, the guiding principle of which is empathy. Such an approach ensures patient-centered care and serves as a powerful source of hope and motivation to actively participate in the treatment process and provides guidance for managing cancer-related emotions. The results of our study show that faith enhances empathy in clinicians in the oncology field, which was reported by 59% of women and 63% of men participating in our survey. However, it was not reported as an effective strategy for challenges related to patients’ deaths or, for instance, breaking bad news interventions in the group with the longest employment time in oncology-related work environment.

Working with oncology patients, despite the challenges and difficulties, gives great professional satisfaction, which is motivated on the one hand by the possibility of helping others in the face of their suffering, and on the other hand by the importance of this support. This professional role, also allows radio-oncologists to express their faith through their intentional and practical caring of others. Clinicians’ participation in building and shaping the identity of the staff is of relevance, as is the sense of mission, as well as signs of respect and recognition among both staff and patients, and their relatives. Clinical professionals with the greatest experience know their value, the value of their work, and are able to derive positive emotions from this satisfaction, which when juxtaposed with the hardships of daily work becomes a source of life and professional wisdom. This wisdom and experience create a balance between life and death, professional and private life, joy from therapeutic successes with an invitation to accompany patients in their final moments.

### Limitations

In the current study, some limitations should be taken into consideration. First, the study group consisted of clinical oncologists specializing in radiotherapy treatments which prevents it from generalization to other clinicians working in oncology settings. Second, the majority of the respondents participating in the study were women which may also impact the obtained results in terms of diversity in beliefs and behavioral aspects. Also, our study did not distinguish between religions which results from the fact that nearly 95% of Poland’s population declare that they belong to a religious denomination, almost 92% are Roman Catholics, and only 3% consider themselves to be non-believers. As an exploratory study, this research did not involve any standardized psychometric testing of participants and relied purely on basic descriptive statistical assessments. In the future such a study warrants a larger cohort of participants and more detailed statistical analysis.

## Conclusions

Communication with patients in oncology-related aspects is a challenging experience and requires a high level of skill from the interlocutors. This study shows that spirituality and religiousness both serve as potential therapeutic strategies for clinicians exposed to patient suffering and death. Religiousness and spirituality can also serve such professionals in managing challenging or negative emotions related to their work. Such interventions, whether in the form of prayer, meditation, communing with art or nature, or provided in any other preferred form, enhance their communication skills and support their abilities to accompany their patients through the difficult journey of coping with cancer.
